# On game-based control systems and beyond

**DOI:** 10.1093/nsr/nwaa093

**Published:** 2020-05-09

**Authors:** Renren Zhang, Fang Wang, Lei Guo

**Affiliations:** Key Laboratory of Systems and Control, Academy of Mathematics and Systems Science, Chinese Academy of Sciences, China; Data Science Institute, Shandong University, China; Key Laboratory of Systems and Control, Academy of Mathematics and Systems Science, Chinese Academy of Sciences, China

## Abstract

This perspective first expounds on the reasons behind the introduction of the game-based control systems (GBCS), delineates their features, provides some examples, and put forward some research problems. The paper then points out that more complex regulation problems on the now rapidly developing man-machine integration systems may also be investigated by expanding the scope of the GBCS.

In the traditional frameworks of both classical and modern control theories, the objects or plants to be controlled or regulated are essentially of ‘passive’ nature, in the sense that they are usually modeled by physical laws and are assumed to have no objectives or payoffs to pursue actively by themselves as in the control of e.g. a car, an aircraft or a chemical process, etc. This may not be the situation, however, for many practical regulation problems in, for example, social, economic and future ‘intelligent’ engineering systems. Such problems share a common characteristic where the objects to be controlled or regulated usually consist of multiple active agents, whose behaviors are not only driven by physical laws, but also by their own interests or objectives. In general, the agents’ interests or objectives may not be the same as those of the global objective of control or regulation, and therefore may result in strategic behaviors of the agents. To regulate such systems, neither traditional game theory nor traditional control theory could be applied directly, and it turns out to be necessary to incorporate a game-based structure in the control theoretical framework [1], though there are some known connections between the two theories [2].

This new class of control system may be referred to as a game-based control system (GBCS) [3,4]. A GBCS has a hierarchical decision-making structure: one regulator at the macro-level and multiple interacting agents at the micro-level. The regulator can be regarded as a global controller that makes decisions first, and then the agents try to optimize their own performances or objectives respectively. We remark that the regulator may contain multiple regulatory variables including either feedforward-feedback control signals or adjustable performance indices or both. Moreover, if the states of the agents can reach a Nash equilibrium as a result of non-cooperative dynamic game, then the control of the GBCS may be regarded as the control of the Nash equilibrium. On the other hand, if the states of the interacting agents are out of balance due to some reason, then the regulation of the GBCS may help to reach the desired balanced state. We would like to point out that the difference between the GBCS and the Stackelberg game is the same as that between control theory and optimization theory. In fact, there are many more problems in the GBCS that need to be investigated as control systems, for example, feedback and uncertainty, controllability, observability, stabilizability, system identification, adaptive control, robust control, etc.

It goes without saying that GBCSs are ubiquitous, and we just briefly mention two examples here. One example is the regulation of macro-economics [5], where a government or organization may exert regulations through policies for various reasons, e.g. the monetary and fiscal policies of the European Union [6], which may be modeled and analysed by using a GBCS. Another example comes from contact robots which can work with and assist human users, such as rehabilitation robots. It has been shown that humans interact with their environments by minimizing both the performance errors and the costs of their actions [7], and the Nash equilibrium arises naturally in human–robot interactions [8]. Since Nash equilibrium strategies may lead to system instability, an external regulator is required, which may be modeled as a stabilization problem of the GBCS.

Preliminary theoretical studies on the GBCS were initiated [1,3,4], and some necessary and sufficient conditions on the controllability of linear GBCSs are provided [3,4]. The study of GBCSs is still in its infancy and more in-depth research is needed. Here we only mention a few interesting problems: whether or not there is a canonical form for GBCSs, how to control GBCSs under uncertainties in structure and information and how mechanism design methods [9] may be used in GBCSs.

Of course, there are many important regulation problems beyond the scope of the GBCS. For example, breakthroughs in information technology and artificial intelligence (AI) are rapidly changing the structure of human society, where there are plenty of emerging and challenging regulation problems. AI has developed from auxiliary tools to good consultations, and may even gradually become a basic social element that is autonomous and should be beneficial to humans. Understanding the behavior of AI systems is essential to our ability to control their actions, reap their benefits and minimize their harms, and thus the study of machine behaviors has been regarded as an emerging field [10]. Furthermore, the objects of social regulation are being upgraded into powerful AI as well as organizations and individuals with capabilities significantly enhanced by AI, and the environment is being transformed into complex systems of man–machine integration. The currently developing intelligent transportation systems, industrial internets, electronic commerce and smart cities are a few examples. In the face of various uncertain risks and challenges in man–machine integration systems (MMIS), such as widespread information collection and integration, complex and highly interconnected heterogeneous subjects, technological difficulties of ethical and legal reconstruction, and the task of algorithm regulation [11], it is of great importance to build a social regulatory system that conforms to such value objectives as safety and stability, equity and justice, democracy and freedom, social welfare and harmonious evolution, etc.

The key to the regulation of MMIS is to regulate the behaviors of humans and machines as well as the emergent behaviors resulting from their interactions and integrations. There are some basic principles that can be followed. For example, being people-oriented, advantageous complementariness, proportionality and algorithm regulation, which means insistence that AI systems should support human institutions and fundamental rights to achieve a fair society; reasonable allocation of human and AI functions to give full play to the complementary advantages of human and artificial intelligence; insistence on the minimum infringement and the balance of legal interests, distinguishing the risk level and determining the regulatory intensity and subjects; and regulating the impact of algorithms from both inside and outside, and promotion of sustainable and harmonious development. Some related issues may be found in various plans, guidelines and principles for the development of AI, see Refs [12] and [13] for two recent ones.

A possible path to regulate MMIS is to combine control theory for engineering systems with the science of law for social systems, or to expand the scope of the above-discussed GBCS to include legal norms as necessary tools for regulation at different levels. This may likely enable us to secure basic ethical and legal issues, and at the same time to realize the technical objectives of regulation for MMIS. There is no doubt that the regulation of MMIS will be of great importance with grand challenges in the future.
